# Grade Expectations: Rationality and Overconfidence

**DOI:** 10.3389/fpsyg.2017.02346

**Published:** 2018-01-12

**Authors:** Jan R. Magnus, Anatoly A. Peresetsky

**Affiliations:** ^1^Department of Econometrics and Operations Research, Vrije Universiteit Amsterdam, Amsterdam, Netherlands; ^2^Tinbergen Institute, Amsterdam, Netherlands; ^3^Department of Applied Economics, National Research University Higher School of Economics, Moscow, Russia

**Keywords:** rational expectations, classroom experiment, overconfidence, education, behavioral education, gender difference, persistence, A22, I21, C91, D01, D90

## Abstract

Confidence and overconfidence are essential aspects of human nature, but measuring (over)confidence is not easy. Our approach is to consider students' forecasts of their exam grades. Part of a student's grade expectation is based on the student's previous academic achievements; what remains can be interpreted as (over)confidence. Our results are based on a sample of about 500 second-year undergraduate students enrolled in a statistics course in Moscow. The course contains three exams and each student produces a forecast for each of the three exams. Our models allow us to estimate overconfidence quantitatively. Using these models we find that students' expectations are not rational and that most students are overconfident, in agreement with the general literature. Less obvious is that overconfidence helps: given the same academic achievement students with larger confidence obtain higher exam grades. Female students are less overconfident than male students, their forecasts are more rational, and they are also faster learners in the sense that they adjust their expectations more rapidly.

## Introduction

Most people overestimate their abilities. Svenson (1981), for example, estimates that 93% of US drivers and 69% of Swedish drivers consider their driving skills “above the median.” Overconfidence appears to be one of the most robust findings in experimental psychology (De Bondt and Thaler, 1995). We contribute to this literature by asking how well undergraduate students forecast their grade, given their ability and other control variables.

The first, perhaps, to investigate this issue was Murstein (1965) using a sample of 76 students from a course in educational psychology at Louisiana State University. Persistency of overconfidence was found, especially for the weaker students. Grimes (2002) studied a sample of 253 students enrolled in a principles of macroeconomics course at Mississippi State University, and found a high degree of overconfidence. Nowell and Alston (2007) used data from a survey conducted in 32 courses, representing every class offered by the economics department at Weber State University in Utah during one semester. The sample consisted of 715 students with a 70% response rate. The authors found that male students with a lower grade point average (GPA) have greater overconfidence; that students in upper division classes have less of a tendency to overestimate their grades relative to students taking lower division courses; that gender matters; and that increasing the importance of tests reduces overconfidence. Hossain and Tsigaris (2015) considered students in a second year statistics for business and economics course at Thompson Rivers University in Kamloops, British Columbia (Canada). A total of 169 students were surveyed with a response rate of over 90%. Students were asked to make several forecasts of their final exam grade during the course. The rational expectations hypothesis was rejected. Expectations move closer to the realized grade as students receive new information on their actual performance closer to the exam. Many other papers reject the hypothesis of rational expectations and confirm student grade overconfidence; see Kruger and Dunning (1999), Svanum and Bigatti (2006), Andrews et al. (2007), Burns (2007), Jensen and Moore (2008), Khachikian et al. (2011), Hossain and Tsigaris (2013), Feld et al. (2017), Foster et al. (2017), Serra and DeMarree (2016), and Sturges et al. (2016).

Is overconfidence helpful or harmful for the student? There is no consensus. Overconfidence may induce a student to allocate less time to study, resulting in poor exams grades. On the other hand, Ballard and Johnson (2005) argue that expectations could become self-fulfilling, possibly because the student with higher expectations will work harder and more intensely on the course. They found that expected grades relate positively to a student's performance in class. Johnson and Fowler (2011) argue, along similar lines, that overconfidence may increase ambitions, morale, resolve, persistence, and thus actually increase the probability of success.

Does overconfidence depend on gender? Are women better forecasters? Again, there is no consensus. Guzman (2012) demonstrated that in the housing market gender is a significant factor in price expectations. Women are less optimistic (perhaps more realistic) about housing prices than men. Also, women tend to be better forecasters of unemployment and inflation than men, also when one controls for income, education, race, age, marital status, number of children in the household, et cetera. Lundeberg et al. (1994) concluded from a sample consisting of three psychology courses containing 70 men and 181 women that both men and women tend to be overconfident, but men more so, especially when they are wrong! Nowell and Alston (2007) analyzed a sample of students enrolled in economics and quantitative courses. They concluded that men were 9% more likely to overestimate their grade than women. Jakobsson (2012) also found a gender difference in the prediction error of exam grades from a sample of 98 students in introductory macroeconomics at Karlstad University (Sweden). Others do not find significant differences in prediction accuracy between men and women. Maxwell and Lopus (1994) reported that both men and women tend to overstate their grade point averages, but they did not find a difference by gender. Grimes (2002) and Andrews et al. (2007) also did not find gender differences in the forecast error. To the best of our knowledge, there is only one paper which states the opposite: Sharma and Shakeel (2015) considered students in India and found that the male students seemed to be more modest in the prediction of their exam grades than the female students.

How persistent are overly optimistic expectations? Do students adjust their forecasts? Murstein (1965) found persistency of overconfidence, especially for the weaker students. The vast majority of the strong students showed no significant change in their predictions as their grade experience accumulated. They believed that they deserved high grades and they received high grades. The weaker students did not change their predictions either, although they should have. In a sample of 60 students from a course in research methods in the Department of Psychological Sciences at Texas Tech University, Serra and DeMarree (2016) concluded that students' predictions of their grades were persistently overconfident because their predictions were biased by their desired level of performance. Foster et al. (2017) experimented with 13 consecutive (weekly) exams in an introduction to educational psychology course. They also found that students did not adjust their expectations. Grimes (2002) and Burns (2007) concluded that students' grade expectations became more accurate as they gained experience in the course. Grimes (2002) noted that women appeared to be more successful in bringing their expectations in line with their performances than men.

The main focus of our paper is the expectation of the student about his or her grade, and the papers discussed above shed some light on what others have found. There exists a vast literature on the broader subject of overconfidence, both from a theoretical and an empirical viewpoint. The bias in social comparative judgments is discussed in comprehensive reviews by Chambers and Windschitl (2004) and Windschitl and O'Rourke Stuart (2015). Moore and Schatz (2017) distinguished between overestimation (what we study), overplacement and overprecision, and they distinguished between two possible benefits of overconfidence: intrapersonal and interpersonal. Armor et al. (2008) argued that “if people believe, rightly or wrongly, that unrealistic optimism has some value, then optimistic bias may be usefully understood as being consistent with people's values and beliefs.” Armor and Taylor (1998) review about 300 theoretical papers on the usefulness of optimism.

In our study we do not discuss a theoretical framework of our findings. We simply introduce a model which helps us to measure overconfidence in students' forecasts. Our aim is to contribute to the questions raised above by analyzing students enrolled in a second-year undergraduate course in statistics at ICEF, Moscow, in total 592 students. During the course each student took three exams and at each exam they forecasted their grade. We address the following research questions: Are students' expectations rational? Are they overconfident? If so, is the level of overconfidence the same for male and female students? Is overconfidence helpful? Do students adjust their exam grades during the course when more information becomes available? And, if so, does the speed of adjustment depend on gender? We find that, in general, students are overconfident, especially male students; overconfidence is helpful; students adjust their forecasts with their experience of the course; and female students adjust their beliefs faster than male students.

The paper is organized as follows. The setup is described in section Methods and Participants. Rationality, overconfidence, and persistence are investigated in section Results. Section Discussion and Conclusions offers some discussion and concluding remarks.

## Methods and participants

### Course organization and grading

The International College of Economics and Finance (ICEF) in Moscow was established in 1997 jointly by the London School of Economics and Political Science (LSE) in London and the Higher School of Economics (HSE) in Moscow. The college offers a 4-year bachelor's program, which is considered to be one of the top programs in economics in Russia. Each year about 200 students enter the program, typically immediately after high school. In their first year the students follow, among other subjects, a course called Statistics-1, and in their second year they follow Statistics-2. Both courses are compulsory. Our data are obtained from students following Statistics-2 over a 5-year period, 2011–2015. In total, 964 students took this course during these 5 years.

In Statistics-2 students take three exams every year, at the end of October (exam 1), the end of December (exam 2), and the end of March (exam 3). The exams are written exams, not multiple choice, and each consists of two parts (80 min each) with a 10 min break between the two parts. The level of the exam questions is the same in the two parts. In order to avoid cheating, students are not allowed to leave and come back during each part of the exam. At the end of part 1 and at the end of the exam the examiner collects each student's work. Each part is graded out of 50 points.

In addition, students have weekly homework assignments although these are not compulsory. All handed-in assignments are graded (out of 100). The variable *HW* denotes for each student the sum of all assignments' grades divided by the total number of assignments. For example, if a student hands in 20 of the 25 assignments and scores 100 (the maximum) for each, then *HW* = (20·100)/25 = 80.

After completion of the three course exams, students take two additional exams (some only take one) in early May administered by the University of London, called *STAT*_1_ and *STAT*_2_. These, like the other exams, are also graded with a maximum score of 100. The final grade *G*_*tot*_ for the course is then determined as

$$\begin{array}{l} G_{tot}=0.14HW+0.14G_{1}+0.14G_{2}+0.21G_{3}\\ \;+0.37\max(STAT_{1},STAT_{2}), \end{array}$$

where *G*_*j*_ is the grade obtained in exam *j*.

Students fail if *G*_*tot*_ is smaller than some threshold to be determined by the teacher, but lying between 32 and 37. Student also fail if *G*_3_, the grade in the third exam, is <25. Exam 3 thus plays a special role in two ways: its weight is higher than for the other two exams and there is a threshold grade of 25.

### Self-assessment

At the end of the first part of each of the three exams each student was asked to forecast (out of 100) his/her grade for this exam (the two parts together). Students were told that answering this question is voluntary: they can answer or they can skip the question. They were also told that their answers could be used for research purposes anonymously. At the moment when the student writes down the forecast he/she knows the questions and his/her answers in part 1, but the student does not yet know the questions of part 2. To encourage students to fill in their forecast and to actually try their best, a bonus is promised. If the difference between the forecast and the grade is ≤ 3 in absolute value, then one bonus point is added to the grade. For example, if the forecast is 49 and the grade is 52, then the grade for this exam is marked up to 53. This procedure had to be and has been approved by the ICEF administration. As a result of the procedure and the possibility of a bonus, the response rate was extremely high (97%). The idea of giving each student an incentive to express his/her opinion was also used in a recent experiment by Blackwell (2010), where students were asked to assess the difficulty of an assignment by guessing the class average, earning a bonus if their guess was close enough.

Smart (or risk averse) students utilize this bonus in the third exam, where the grade must be ≥25. If the student chooses the forecast *F*_3_ such that 21 ≤ *F*_3_ ≤ 27, then a grade *G*_3_ = 24 would be marked up to 25. Some students actually do this, but they then typically choose *F*_3_ = 24 or 25 and not, say, 21 or 27. The special role of the third exam and the overrepresentation of 24 and 25 in the sample of third exam forecasts have to be taken into account when we do our statistical analysis, and we shall discuss this issue further below.

### The data

The data consist of the grades *G*_*j*_ and the forecasts *F*_*j*_ (*j* = 1, 2, 3) for each of our students, and our interest is focused on the excess expectation in exam *j*:

(1) $$D_{j}=F_{j}-G_{j}.$$

We have some background knowledge on each student, namely the grades of the first-year calculus (*calc*) and first-year statistics (*stats*) exams, the grade point average at the end of the first year (*gpa*), and whether the student is male or female (*female* = 1 for women and 0 for men). We also know in which year the exam took place (*year* = 2011, 2012, 2013, 2014, 2015).

For the homework assignments we know for each student how many assignments the student handed in (*nhw*_*j*_), the sum of the grades per exam period (*shw*_*j*_), and the number of handed-out assignments (*nhwmax*_*j*_). The index *j* now refers to a period rather than to a point in time: *j* = 1 refers to the period up to the first exam, *j* = 2 to the period between the first and second exams, and *j* = 3 to the period between the second and third exams. The number of handed-out assignments (*nhwmax*_*j*_) may vary from year to year, in fact from 4 to 7 in period 1, from 6 to 7 in period 2, and from 9 to 12 in period 3.

From these “raw” data we can compute the ratios

(2) $$rnhw_{j}\;=\;nhw_{j}/nhwmax_{j},\;rshw_{j}\;=\;shw_{j}/nhw_{j},$$

which denote, respectively, the relative number of submitted assignments for each student in period *j* (0 ≤ *rnhw*_*j*_ ≤ 1), and the average submitted assignment grade for each student in period *j* (0 ≤ *rshw*_*j*_ ≤ 100), where we set *rshw*_*j*_ = 0 if *nhw*_*j*_ = 0. These ratios will be used later in the analysis.

In order to obtain a clean and complete sample, some data screening was necessary. Of the original 964 students we excluded those students who (a) did not take all three exams; or (b) had repeated the first year; or (c) had failed the course last year; or (d) had taken a break between the first and second year. This left us with 840 students.

Of these 840 students, a further 248 were excluded because they did not provide all three forecasts or we didn't have their first-year results. As a consequence, 592 students remain on which we have complete information. The results of the University of London exams are not used in our analysis.

A summary of these “raw” and basic data is provided in Table 1 (Kernel density plots of the basic data are provided as Supplementary Material). There is substantial variation in the exam grades *G* and the forecasts *F* over the years, possibly because the difficulty of the exams varies (although the same instructor taught the course over this period) or the quality of the student population varies (because of changes in admission policies). This suggests that year dummies may be important. On the whole it seems that students are too optimistic about their abilities, because *F*_*j*_ > *G*_*j*_ occurs more frequently than *F*_*j*_ < *G*_*j*_. Also, in the first two exams large deviations occur where |*F*_*j*_ − *G*_*j*_| can be larger than 10, in contrast to the third exam where the deviation is much smaller. This suggests that students learn from their past forecast errors.

**Table 1:** Basic data, averaged.

**Year**	**2011**	**2012**	**2013**	**2014**	**2015**	**All**
*G*_1_	40.81	33.87	33.94	40.18	28.13	35.30
*G*_2_	48.41	34.49	47.50	27.35	32.70	36.90
*G*_3_	47.77	41.41	39.00	38.16	38.55	40.27
*F*_1_	36.49	43.04	39.06	34.68	38.93	38.21
*F*_2_	48.82	42.85	40.54	38.06	38.58	40.98
*F*_3_	48.49	37.86	41.69	38.00	37.41	40.06
Observations	79	103	129	158	123	592
*female* (%)	40.51	36.89	41.09	39.87	47.15	41.22

From these raw data it is not immediately clear what the answers are to our questions. To achieve this we need more sophisticated statistical techniques than simple averages. In the next section we will address each of our research questions in turn and develop the required models as we progress.

## Results

### Rationality

Our first question is whether our students have rational expectations about their exam grades. In the Introduction we mentioned some literature where it is found that students overestimate their abilities, that is, that they are not rational. If we also find this (as we shall) then a second question arises, namely whether male and female students are equally irrational or that perhaps female students behave more rationally than male students.

Our experimental data differ from the data in most papers in three respects: first, we use a 0–100 grade system, while other papers typically use the more discrete F-D-C-B-A (0–4) grade system; second, our students make their forecast after they have already finished half the exam; and third, our students have a real incentive to make their forecast as precise as possible, as they get a bonus for an accurate forecast.

There is, in addition, one other feature of our data, namely the fact that we collected exam results and the associated forecasts during 5 years (2011–2015). We know from the previous section that the exams are not equally difficult in each year, and these discrepancies need to be taken into account. Thus, following Hossain and Tsigaris (2015), we regress the excess expectation *D*_*j, i*_ = *F*_*j, i*_ − *G*_*j, i*_ for student *i* in exam *j* for each of the three exams separately, and include year dummies *year*12_*i*_ − *year*15_*i*_. The regression then reads

(3) $$D_{j,i}=\alpha_{j}+{x^{\prime }}_{j,i}\beta_{j}+yea{r^{\prime }}_{i}\gamma_{j}+female_{i}\delta_{j}+\varepsilon_{j,i},$$

where *x*_*j, i*_ is the vector of all available information at the time of exam *j* of the *i*-th student's previous academic achievements, and the control variables are a vector of time dummies

$$year_{i}=\left(year12_{i},year13_{i},year14_{i},year15_{i}\right)\prime ,$$

and the female/male dummy *female*_*i*_. The α_*j*_, β_*j*_, γ_*j*_, and δ_*j*_ are unknown parameters (parameter vectors), and ε_*j, i*_ is the random error, which we assume to be independently and identically distributed with mean zero.

We define a student to be rational when the conditional expectation E(*D*_*j, i*_|*x*_*j, i*_) = 0 and this translates to testing the null hypothesis

$${\text{H}}_{0}:\beta_{j}=0$$

for each of the three exams *j* = 1, 2, 3. Note that the dimension of β_*j*_ is not the same for each *j*, because more information is available at the second exam than at the first exam, and even more information is available at the third exam. In fact, the dimension is 5 at the first exam (*calc, stats*, *gpa*, *rnhw*_1_, *rshw*_1_), 8 at the second (the previous plus *G*_1_, *rnhw*_2_, *rshw*_2_), and 11 at the third (the previous plus *G*_2_, *rnhw*_3_, *rshw*_3_).

Recall that the third exam is special because students fail the course if *G*_3_ < 25. From the student's point of view it makes good sense to predict 21 ≤ *F*_3_ ≤ 27, because then (and only then) a grade *G*_3_ = 24 will be marked up to 25. This is “rational” behavior, but not according to our definition. In practice, these students choose *G*_3_ = 24 or 25, but almost never 26 or 27. Also, some students are confused and believe that this rule applies to all three exams and not only to the third. To avoid these problems we only include forecasts which satisfy *F*_*j, i*_ > 25. We recognize that this censoring could (slightly) shift the data so that finding overconfidence becomes more likely. Since this is something that the data will not reveal, we need to assume that such a shift does not take place.

Table 2 contains the regression results for each of the three exams separately. The last row in the table contains the *p*-value of the *F*-test used for testing the hypothesis that β_*j*_ = 0. The reported *p*-values are < 0.2% thus rationality is firmly rejected in this model. The *p*-value is lowest at the first exam, still very low at the second (where more information is available), and much higher (but still < 0.2%) at the third (where even more information is available). More information thus leads to more rational decisions, which is not as obvious as it may seem, because too much information might turn into confusion and lead to less rational behavior. This, however, does not happen here.

**Table 2:** Rationality, *F*_*j*_ > 25.

	***D*_1_**	***D*_2_**	***D*_3_**
*calc*	−0.106	−0.110	0.138
	(0.097)	(0.093)	(0.097)
*stats*	−0.159	−0.247^**^	−0.119
	(0.137)	(0.118)	(0.133)
*gpa*	−0.155	0.143	−0.086
	(0.163)	(0.141)	(0.153)
*rnhw*_1_	−0.773	−0.515	1.811
	(3.028)	(3.335)	(3.690)
*rshw*_1_	−0.055	−0.031	0.086^*^
	(0.041)	(0.039)	(0.045)
*G*_1_		0.121^**^	0.141^**^
		(0.054)	(0.058)
*rnhw*_2_		−8.071^**^	−0.747
		(3.203)	(3.929)
*rshw*_2_		−0.032	0.002
		(0.041)	(0.049)
*G*_2_			−0.162^***^
			(0.060)
*rnhw*_3_			−5.741
			(3.486)
*rshw*_3_			−0.037
			(0.046)
*female*	−4.913^***^	−3.060^**^	−2.373^*^
	(1.381)	(1.224)	(1.293)
constant	31.52^***^	18.22^***^	8.65^*^
	(4.71)	(4.21)	(4.69)
Time dummy	Yes	Yes	Yes
Observations	414	458	393
*R*^2^	0.326	0.296	0.126
$R_{adj}^{2}$	0.309	0.275	0.088
RMSE	13.50	12.48	12.10
*p*-val (*F*-test)	0	1.9·10^−7^	0.0011

Standard errors in parentheses;

*** *p < 0.01*,

** *p < 0.05*,

* *p < 0.1*.

Some preliminary conclusions can be drawn from Table 2. First, it seems that good students (high marks in *calc*, *stats*, and *gpa* in the previous year) are more cautious than not so good students in their predictions, at least for the first exam. If the student does well in the first exam, then he/she becomes less cautious and in fact tends to overpredict the results of both the second and the third exam. Having learnt their lesson, students become more cautious again: doing well in the second exam leads to more rather than to less caution in predicting their mark for the third exam. There is a big impact of gender. Women are more cautious than men in all three exams, although the impact diminishes over time.

Kruger and Dunning (1999) concluded that low-performing students significantly overestimate their performance, while high-performing students are more accurate in their forecasts. Feld et al. (2017) pointed out that this effect could at least partially be explained by measurement errors. They showed that after correction for measurement errors (using instrumental variables) the Dunning-Kruger effect is still observed, but significantly weaker than before the correction.

To further investigate the difference in rationality between women and men, we also estimate the extended model.

(4) $$D_{j,i}=\alpha_{j}+{x^{\prime }}_{j,i}\beta_{j}+yea{r^{\prime }}_{i}\gamma_{j}+\varepsilon_{j,i},$$

for men and women separately. The results are presented in Table 3, where we have again excluded all students with *F*_*j, i*_ ≤ 25.

**Table 3:** Rationality, men vs. women, *F*_*j*_ > 25.

	**Men**			**Women**		
	***D*_1_**	***D*_2_**	***D*_3_**	***D*_1_**	***D*_2_**	***D*_3_**
*calc*	−0.152	−0.114	0.032	−0.087	−0.089	0.222
	(0.135)	(0.128)	(0.129)	(0.142)	(0.139)	(0.157)
*stats*	−0.084	−0.265	−0.230	−0.277	−0.239	−0.062
	(0.194)	(0.160)	(0.171)	(0.189)	(0.176)	(0.224)
*gpa*	−0.092	0.282	0.166	−0.222	−0.028	−0.308
	(0.232)	(0.193)	(0.197)	(0.224)	(0.210)	(0.254)
*rnhw*_1_	−1.473	0.392	6.139	2.447	−2.430	−6.787
	(4.111)	(4.285)	(4.482)	(4.716)	(5.800)	(6.827)
*rshw*_1_	−0.071	−0.033	0.017	−0.035	−0.023	0.147^**^
	(0.058)	(0.053)	(0.059)	(0.057)	(0.059)	(0.070
*G*_1_		0.0521	0.214^***^		0.207^**^	0.047
		(0.075)	(0.078)		(0.081)	(0.095)
*rnhw*_2_		−9.627^**^	−1.213		−5.095	4.109
		(4.104)	(4.831)		(5.467)	(7.193)
*rshw*_2_		0.006	0.052		−0.090	−0.106
		(0.053)	(0.060)		(0.066)	(0.091)
*G*_2_			−0.240^***^			−0.001
			(0.074)			(0.108)
*rnhw*_3_			−10.776^**^			0.281
			(4.419)			(5.826)
*rshw*_3_			0.019			−0.102
			(0.058)			(0.083)
Constant	28.38^***^	11.40^**^	3.48	30.83^***^	23.76^***^	16.26^**^
	(6.75)	(5.79)	(6.15)	(6.62)	(6.32)	(7.46)
Time dummy	Yes	Yes	Yes	Yes	Yes	Yes
Observations	244	268	234	170	190	159
*R*^2^	0.251	0.302	0.140	0.418	0.301	0.164
$R_{adj}^{2}$	0.222	0.269	0.0812	0.386	0.253	0.076
RMSE	14.62	13.21	12.14	11.85	11.41	11.99
*p*-val (*F*-test)	3.6·10^−5^	0.0060	0.0060	1.4·10^−7^	0.0002	0.0434

Standard errors in parentheses;

*** *p < 0.01*,

** *p < 0.05*,

* *p < 0.1*.

By including the *female* dummy (as in Table 2) we distinguish between men and women, but only by allowing the level to change from α_*j*_ for men to α_*j*_ + δ_*j*_ for women. By separating men and women (as in Table 3, now of course without the *female* dummy), we also allow the β_*j*_-coefficients to be different.

Our preliminary conclusions still hold in this extended framework. Women are more cautious than men. Good students are more cautious than not-so-good students in their predictions, at least for the first exam. If the student does well in the first exam, then he/she becomes too optimistic in predicting the second exam, but doing well in the second exam does not lead to such optimism. The *p*-values are higher than in Table 2 but still well under 0.1%, except for women in the third exam where the *p*-value is close to 5%. We thus find that our female students became more rational in the third exam, while men continue to exhibit irrational behavior.

It is often thought that women behave more rationally than men, and this is indeed what we find. But there is no consensus in the literature. Ballard and Johnson (2005) reported that gender is a significant determinant of student expectations: women in an introductory microeconomics course expected a grade that was one-fourth of a letter grade (0.25 on a 4.0 scale) lower than the grade expected by the men. However, after controlling for expectations and secondary-schooling experience with economics, the gender effect became small and insignificant. Hossain and Tsigaris (2015) also found that gender makes no difference in this respect.

### Overconfidence

In the previous section we rejected rationality in predicting exam results and we saw that there is a difference between male and female students. Our next step is to try and explain this lack of rationality, and our hypothesis is that students (especially male students) are too confident about their abilities. When a student has more confidence than is justified by his or her grades, we call this student “overconfident”; see i.e., Windschitl and O'Rourke Stuart (2015).

It makes sense that a student who does well in exams gains in confidence. But perhaps the opposite is also true, that is, a confident student-other things being equal-performs better than one lacking in confidence (Ballard and Johnson, 2005; Johnson and Fowler, 2011). In addition to studying overconfidence we also try to answer this somewhat subtler question in the current section.

An overconfident student will produce a forecast which is higher than can be explained by previous academic results. We write the forecast as

(5) $$F_{j,i}=\alpha_{j}+{x^{\prime }}_{j,i}\beta_{j}+yea{r^{\prime }}_{i}\gamma_{j}+\varepsilon_{j,i}$$

which is the same as Equation (3), except that the dependent variable is now the forecast *F*_*j, i*_ rather than the excess expectation *D*_*j, i*_ and also that the *female* dummy has been deleted.

The reason for not including the *female* dummy is that we think of the forecast as a combination of two effects: a part based on factual information and a remainder which we identify with overconfidence. This overconfidence will depend on other things, one of which may be gender. We don't observe the remainder (the errors ε_*i*,*i*_), but we can predict it through the residuals

(6) $$conf_{j,i}=F_{j,i}-{\hat{\alpha }}_{j}-{x^{\prime }}_{j,i}{\hat{\beta }}_{j}-yea{r^{\prime }}_{i}{\hat{\gamma }}_{j},$$

where ${\hat{\alpha }}_{j},{\hat{\beta }}_{j}$, and ${\hat{\gamma }}_{j}$ are the least-squares estimates from Equation (5). These residuals thus capture that part of the student's forecast which cannot be explained rationally, and thus correspond to our idea of (over)confidence, which is why we denote them by *conf*_*j, i*_. Note that if we would include the *female* dummy in Equation (5) then *conf* and *female* would be orthogonal to each other, and this is not reasonable.

Overconfidence, thus defined, may include some information which is not available to us, such as private lessons taken before the exam or certain psychological features of the student. Since this information is not available to us we ignore it.

In the first step of the estimation procedure we thus estimate *F*_*j, i*_ and obtain the residuals *conf*_*j, i*_. In the second step we regress the exam grades *G*_*j, i*_ on the same set of regressors as in Equation (5) and, in addition, on the residuals *conf*_*j, i*_ and the *female* dummy (and a cross term):

(7) $$\begin{array}{l} G_{j,i}=\alpha_{j}+{x^{\prime }}_{j,i}\beta_{j}+yea{r^{\prime }}_{i}\gamma_{j}+female_{i}\delta_{j}+conf_{j,i}\phi_{j}\\ \;+female_{i}\times conf_{j,i}\psi_{j}+\varepsilon_{j,i}. \end{array}$$

The results of the two-step procedure are presented in Table 4. The left panel (columns *F*_1_, *F*_2_, *F*_3_) gives the results of the first step. We see that first-year calculus and (to a lesser extent) statistics are important for *F*_1_, but that home assignments are not important for the forecast. For *F*_2_, the result *G*_1_ of the first exam is important, while first-year calculus remains important as well. For the final exam the forecast *F*_3_ depends much on the results of the earlier two exams *G*_1_ and *G*_2_. The results of first-year calculus (and statistics) are not important anymore; these are absorbed in the grades *G*_1_ and *G*_2_, because recent information is more relevant than older information. We note that $R_{adj}^{2}$ increases with the exam number, suggesting that with each exam students become more accurate in their forecasts.

**Table 4:** Overconfidence results.

	**First step**			**Second step**		
	***F*_1_**	***F*_2_**	***F*_3_**	***G*_1_**	***G*_2_**	***G*_3_**
*conf*				0.225^***^	0.282^***^	0.345^***^
				(0.050)	(0.055)	(0.067)
*female*				1.329	0.750	0.204
				(1.064)	(0.986)	(1.071)
*female × conf*				0.171^**^	−0.00955	−0.187
				(0.087)	(0.097)	(0.116)
*calc*	0.443^***^	0.325^***^	0.083	0.529^***^	0.411^***^	−0.068
	(0.090)	(0.078)	(0.077)	(0.073)	(0.074)	(0.079)
*stats*	0.223^*^	−0.032	0.055	0.377^***^	0.218^**^	0.178
	(0.128)	(0.101)	(0.107)	(0.103)	(0.094)	(0.109)
*gpa*	0.0796	0.123	−0.046	0.270^**^	0.004	0.060
	(0.152)	(0.120)	(0.122)	(0.122)	(0.112)	(0.126)
*rnhw*_1_	−1.074	−0.651	−0.197	0.798	0.287	−2.511
	(2.818)	(2.837)	(2.960)	(2.274)	(2.657)	(3.062)
*rshw*_1_	0.0191	−0.008	0.058	0.072^**^	0.017	−0.030
	(0.038)	(0.033)	(0.036)	(0.031)	(0.031)	(0.037)
*G*_1_		0.305^***^	0.268^***^		0.186^***^	0.122^**^
		(0.046)	(0.047)		(0.043)	(0.048)
*rnhw*_2_		−1.125	−3.813		7.416^***^	−2.436
		(2.723)	(3.150)		(2.547)	(3.221)
*rshw*_2_		0.055	−0.005		0.088^***^	−0.005
		(0.035)	(0.039)		(0.032)	(0.040)
*G*_2_			0.394^***^			0.550^***^
			(0.048)			(0.049)
*rnhw*_3_			1.658			7.836^***^
			(2.797)			(2.857)
*rshw*_3_			−0.012			0.020
			(0.037)			(0.038)
Constant	7.12	14.79^***^	18.08^***^	−24.24^***^	−3.25	10.11^***^
	(4.42)	(3.59)	(3.77)	(3.54)	(3.36)	(3.85)
Year dummy	Yes	Yes	Yes	Yes	Yes	Yes
Observations	414	458	393	414	458	393
*R*_2_	0.353	0.525	0.642	0.719	0.734	0.682
$R_{2}^{adj}$	0.338	0.512	0.627	0.710	0.725	0.667
RMSE	12.67	10.64	9.72	10.13	9.92	9.91

Standard errors in parentheses;

*** *p < 0.01*,

** *p < 0.05*,

* *p < 0.1*.

The right panel (*G*_1_, *G*_2_, *G*_3_) gives the results of the second step and allows us to test various hypotheses. We see that ϕ is significantly positive at the 1% significance level for all three exams. Its value increases with time/exam number (0.225, 0.282, 0.345), so the impact of overconfidence increases; at least for the men. For the women, the impact decreases (0.396, 0.272, 0.158) when we take the cross term into account. There is evidence in the literature that the more important is the exam the smaller is the overconfidence (Nowell and Alston, 2007). In our case this is true for women but not for men.

The *female* dummy is not significant and its cross term with *conf* is significant (at 5%) only for the first exam. In contrast to the results in the left panel (the forecasts), more of the “factual” regressors *x*_*j*_ are significant in the right panel (the grades). The impact of the first-year courses (calculus, statistics, GPA) decreases during the second year, as is to be expected. The grades are significant: *G*_1_ is significant in the *G*_2_ regression (and somewhat less in the *G*_3_ regression), and *G*_2_ is significant in the *G*_3_ regression. Homework results, while not significant for the students' forecasts, are significant for the grades, but only the most recent homework results. The reason, perhaps, is that students understand that homework results are not representative, because there is much collaboration among students and in fact some cheating, so they don't take it into account when forming their forecast. But the plain fact that a student submits the homework (whether own work or not) apparently helps to get a better grade. This finding agrees with Weems (1998), but not with Geide-Stevenson (2009).

Thus we conclude that (a) overconfidence helps in getting a better grade; (b) the impact of information deteriorates quickly over time; (c) homework results are important for the grades, but unimportant for the forecasts; and (d) gender is *not* significant in exam grades.

We next ask: does overconfidence depend on gender? To answer this question we consider the regression

(8) $$conf_{j,i}=\alpha_{j}+female_{i}\delta_{j}+\varepsilon_{j,i},\;(j=1,2,3)$$

where we note that *conf* is orthogonal to the *year* dummy and the available information in *x*, because *conf* is the vector of residuals from Equation (5). Regression results are presented in the Table 5.

**Table 5:** Overconfidence results.

	**(1)**	**(2)**	**(3)**
*female*	−5.036^***^	−2.987^***^	−2.699^***^
	(1.228)	(0.987)	(0.971)
Constant	2.068^***^	1.239^*^	1.092^*^
	(0.787)	(0.636)	(0.618)
Observations	414	458	393
*R*^2^	0.039	0.020	0.019
$R_{adj}^{2}$	0.037	0.018	0.017
RMSE	12.30	10.41	9.45

Standard errors in parentheses;

*** *p < 0.01*,

** p < 0.05,

* *p < 0.1*.

Table 5 shows that, given the same objective factors, male students tend to be more optimistic in forecasting their exam grades than female students. The difference ranges from approximately 2.7–5.0 grade points, and this difference seems to decrease over time (within one exam year). Transforming these grade points to a 0–4 scale we divide by 25 and obtain 0.11 and 0.20, which is of the same order as in Ballard and Johnson (2005).

### Persistence

In the previous section we predicted and studied overconfidence as measured by the residuals *conf*_*j, i*_ for each student *i* and exam *j*. We found that this overconfidence tends to become smaller as the year progresses. We now address this issue in more depth. That is, we ask whether overconfidence decreases, which would mean that students adjust their (over)confidence.

To answer this question we estimate the dynamic regressions

(9) $$conf_{j,i}=\alpha_{j}+yea{r^{\prime }}_{i}\gamma_{j}+\theta_{j}conf_{j-1,i}+\varepsilon_{j,i}.(j=2,3)$$

If |θ_2_| < 1 then learning takes place between exams 1 and 2. Similarly, if |θ_3_| < 1 then learning takes place between exams 2 and 3. We run these regressions separately for men and women, because we have seen that overconfidence is not the same for men and women.

The results are presented in Tables 6, 7. It is clear that adjustment occurs, since |θ_*j*_| is significantly smaller than one. For the adjustment from exam 1 to exam 2 we find θ_2_ = 0.19 for women and θ_2_ = 0.35 for men. The difference between men and women is statistically significant. For the adjustment from exam 2 to exam 3 we find θ_3_ = 0.43 for women and θ_3_ = 0.35 for men, and this difference is not statistically significant.

**Table 6:** Persistence from exam 1 to exam 2.

	**Men**	**Women**
*conf*_1_	0.349^***^	0.185^***^
	(0.054)	(0.062)
*year*12	−1.252	−3.063
	(2.564)	(2.420)
*year*13	−1.956	−1.422
	(2.542)	(2.436)
*year*14	−1.461	−2.097
	(2.494)	(2.252)
*year*15	−0.922	−3.309
	(2.578)	(2.394)
Constant	2.040	0.719
	(2.025)	(1.826)
Observations	206	152
*R*^2^	0.177	0.076
$R_{adj}^{2}$	0.156	0.0448
RMSE	10.31	8.55

Standard errors in parentheses;

*** *p < 0.01*,

**p < 0.05,

*p < 0.1.

**Table 7:** Persistence from exam 2 to exam 3.

	**Men**	**Women**
*conf*_2_	0.351^***^	0.431^***^
	(0.050)	(0.072)
*year*12	−0.431	2.050
	(2.029)	(2.275)
*year*13	2.052	−2.218
	(2.008)	(2.216)
*year*14	1.487	−1.562
	(1.904)	(2.086)
*year*15	1.978	−1.416
	(2.042)	(2.216)
Constant	−0.855	−0.322
	(1.513)	(1.641)
Observations	213	143
*R*^2^	0.201	0.238
$R_{adj}^{2}$	0.181	0.210
RMSE	8.68	7.87

Standard errors in parentheses;

*** *p < 0.01*,

**p < 0.05,

*p < 0.1.

Thus we conclude that (a) confidence adjustment occurs for both male and female students; (b) the adjustment from exam 1 to exam 2 is stronger than the adjustment from exam 2 to exam 3; (c) female students are faster learners, certainly in the step from exam 1 to exam 2; and (d) overconfidence persists (since the values of θ_*j*_ are all positive) and this persistency is stronger for men than for women.

We can go one step further. In the above regressions we estimated the average values of the adjustment coefficient for male and female students. But each student is different and the adjustment coefficient may vary from student to student. In order to estimate the individual values of the adjustment coefficient we model θ_*j*_ as a function of the individual characteristics of a student:

(10) $$\theta_{j,i}=\theta_{0,j}+{x^{\prime }}_{j,i}\theta_{1,j}\;(j=2,3).$$

Inserting Equation (10) in Equation (9) then gives.

(11) $$conf_{j,i}=\alpha_{j}+yea{r^{\prime }}_{i}\gamma_{j}+\theta_{j,i}conf_{j-1,i}+\varepsilon_{j,i}\;(j=2,3).$$

Instead of estimates ${\hat{\theta }}_{j}$ we now obtain distributions (over *i*) of estimates.

$${\hat{\theta }}_{j,i}={\hat{\theta }}_{0,j}+{x^{\prime }}_{j,i}{\hat{\theta }}_{1,j}$$

**Figure 1:**
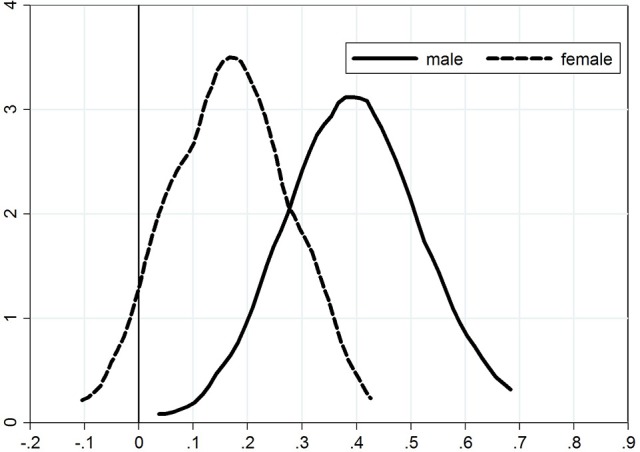
Persistence from exam 1 to exam 2.

**Figure 2:**
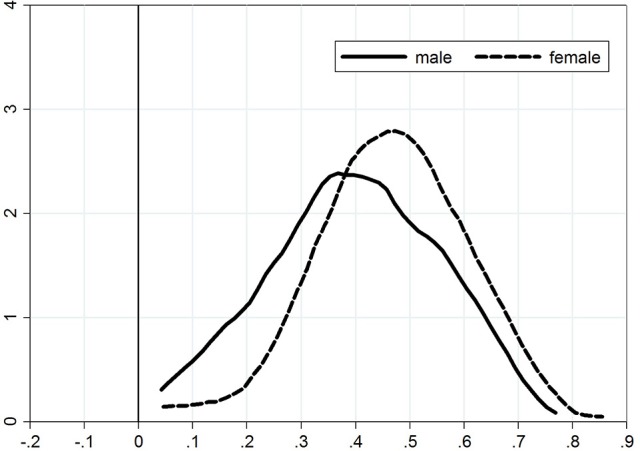
Persistence from exam 2 to exam 3.

Kernel density plots for the distribution of the adjustment coefficient are presented in Figure 1 for the adjustment from exam 1 to exam 2) and Figure 2 for the adjustment from exam 2 to exam 3). We see from the first figure that the density plot for women is shifted toward zero, again demonstrating that female students are faster learners (lower persistence) than male students. There is no significant difference between the density plots for the adjustment from exam 2 to exam 3.

The figures provide further (and more detailed) confirmation of our previous conclusions, namely that (a) adjustment takes place; (b) women are faster learners that men; and (c) there is a persistency of overconfidence from one exam to the next, which is stronger for men than for women.

## Discussion and conclusions

In this paper we studied second-year undergraduate students in a statistics course over a period of 5 years, comparing their grades with their forecasts. As expected, we find that the students' grade expectations are not rational and that most students are overconfident, which is in agreement with the general literature. Our study had the advantage of a relatively large number of students and a high response rate, and thus contributes to various issues (many of them unresolved) in the general area of rationality, overconfidence, and persistence. The following conclusions emerge.

First, overconfidence decreases during the course and is smallest at the third exam, which shows that students adjust their expectations as information accrues (Grimes, 2002; Burns, 2007), in particular when the third exam has a higher weight in the total course grade (Nowell and Alston, 2007). Some studies did not find this adjustment (Murstein, 1965; Serra and DeMarree, 2016; Foster et al., 2017), others did (Grimes, 2002; Burns, 2007). One of the reasons for the discrepancy in the literature may be attributed to the content/essence/nature of the course. Foster et al. (2017) studied the results of 13 consecutive exams in educational psychology, where the content for each exam covers a separate topic. Our course is quite different in that the content is cumulative: the next exam uses concepts from previous parts of the course.

Second, female students have a lower level of overconfidence than male students, thus exhibiting more rational behavior. This is of interest because the literature is not in agreement on this issue. Our results are similar to what Guzman (2012) found in financial forecasts and Lundeberg et al. (1994) and Jakobsson (2012) for grade forecasts. But others (e.g., Maxwell and Lopus, 1994; Grimes, 2002; Andrews et al., 2007) did not find such differences.

Third, female students are not only better forecasters, they are also faster learners than male students, showing a faster adjustment of their grade expectations. We did not find difference in *grades* between female and male students. The role of gender in the fields of science, technology, engineering, and math (STEM) is widely discussed in the literature (see e.g., Schmader, 2002, 2010). Schmader (2002) concluded that when gender identity is not linked to test performance, women perform equally to men, and this finding is confirmed in our study.

Fourth, overconfidence has a positive effect on exam grades. Some studies suggest that overconfident students are less successful at exams since they allocate less time and efforts to study. This may be the case for some students, but we find that for most students overconfidence is advantageous, possibly because it increases ambition, morale, resolve, persistence, and hence the probability of success (Ballard and Johnson, 2005; Johnson and Fowler, 2011).

Finally, a suggestion to teachers based on our findings. Don't wait too long in setting your first test. This will help students to adjust their expectations at an early stage, and this in turn will be of use to them in their allocation of time and effort for the course.

## Author contributions

All authors listed have made a substantial, direct and intellectual contribution to the work, and approved it for publication.

### Conflict of interest statement

The authors declare that the research was conducted in the absence of any commercial or financial relationships that could be construed as a potential conflict of interest.
