# Supplementary figures and images for: Grade Expectations: Rationality and Overconfidence
Source: Front Psychol. 2018 Jan 12;8:2346. doi: 10.3389/fpsyg.2017.02346 (PMC5770583; doi:10.3389/fpsyg.2017.02346)

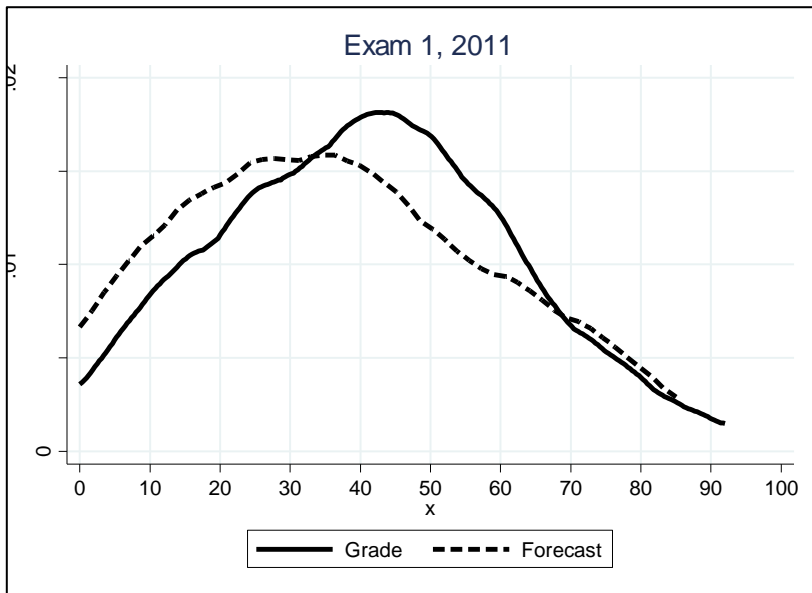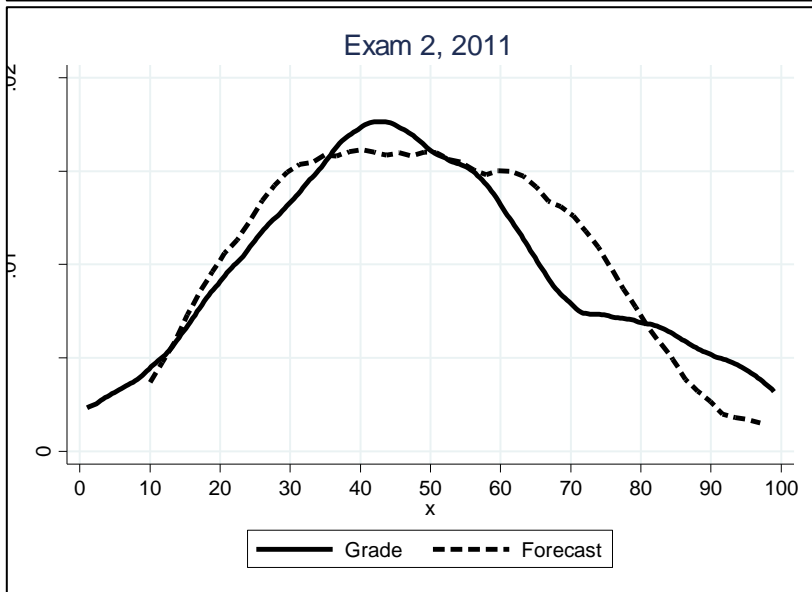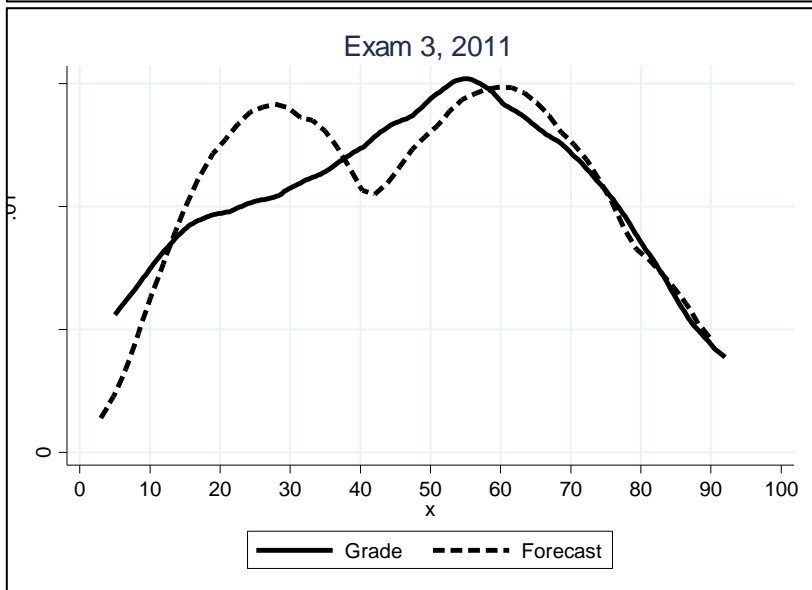

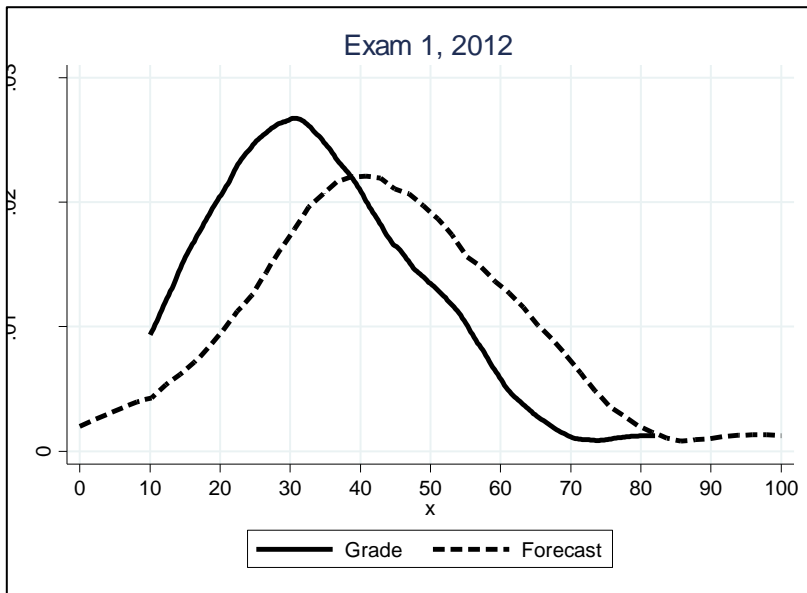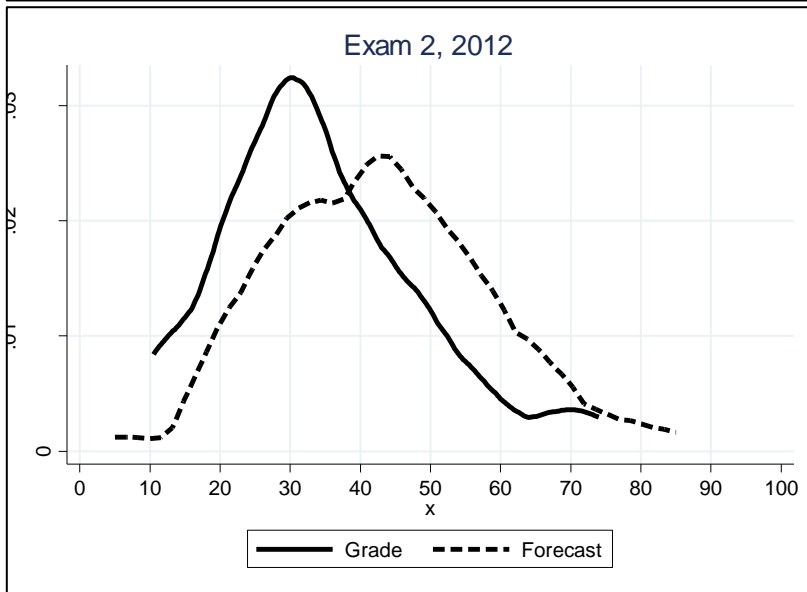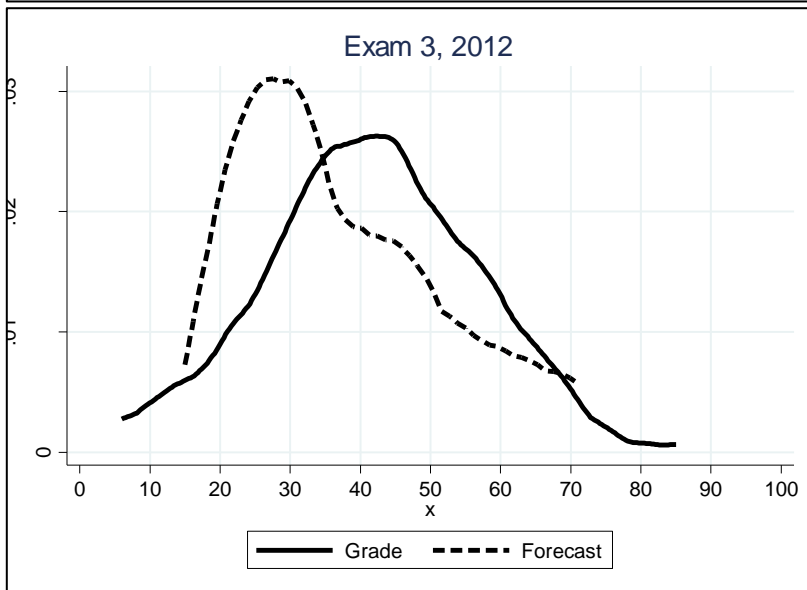

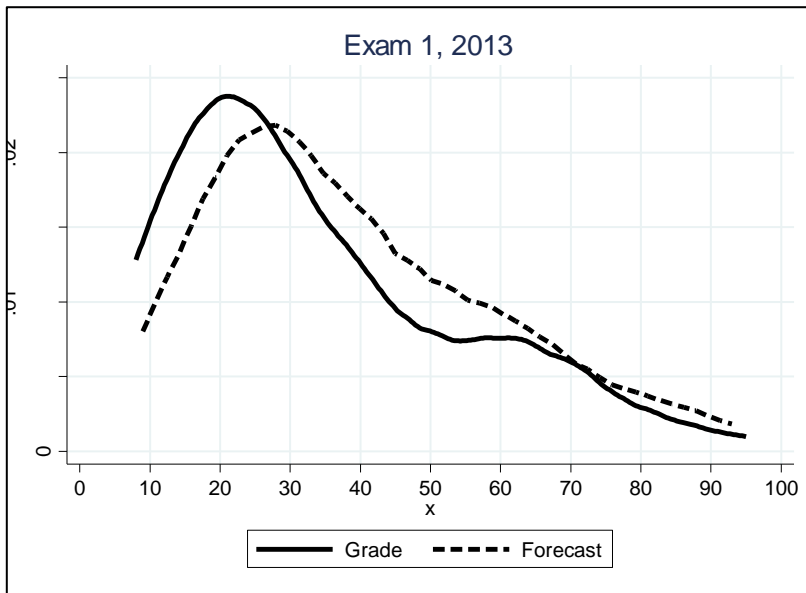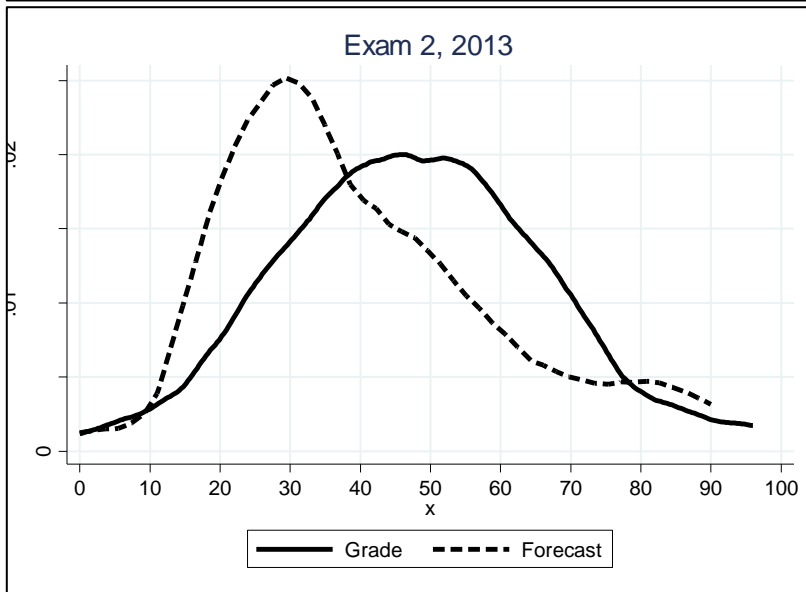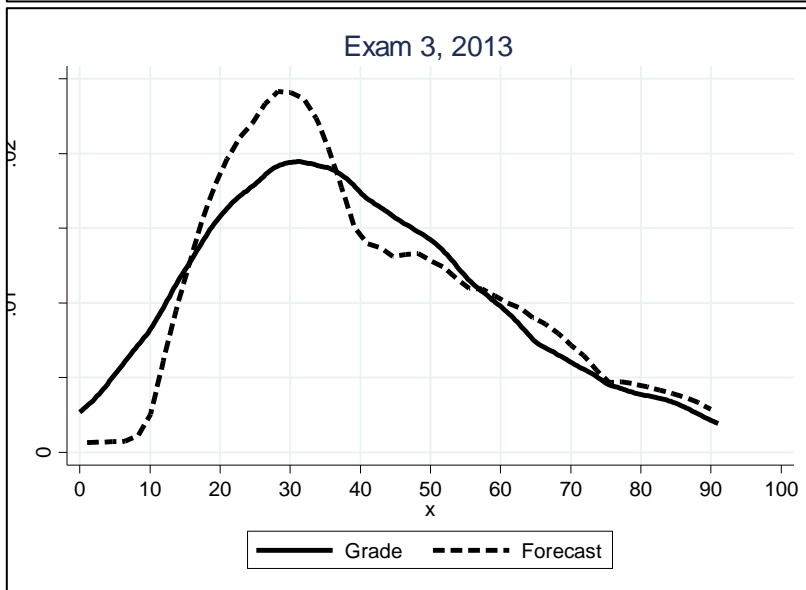

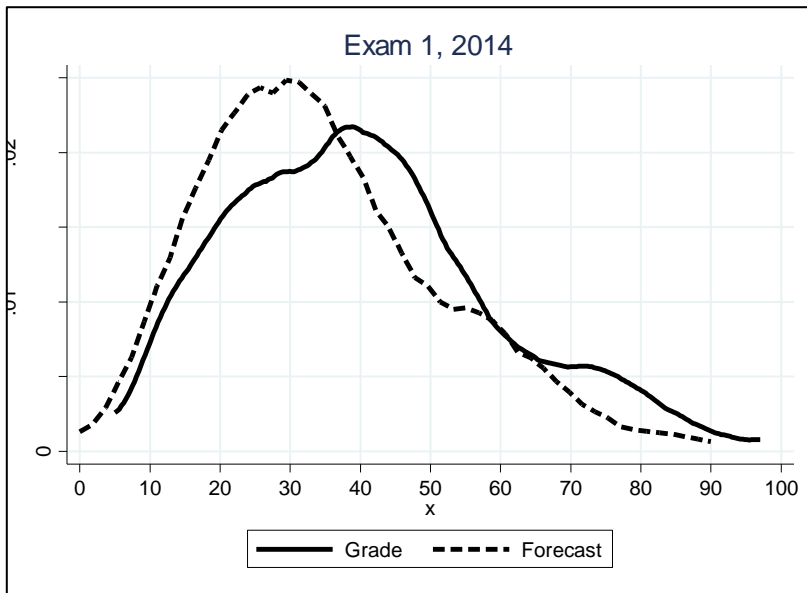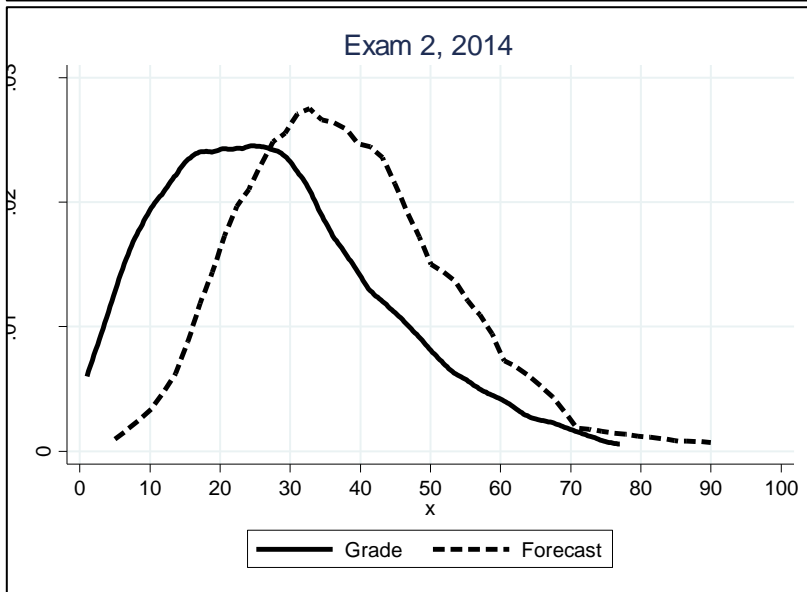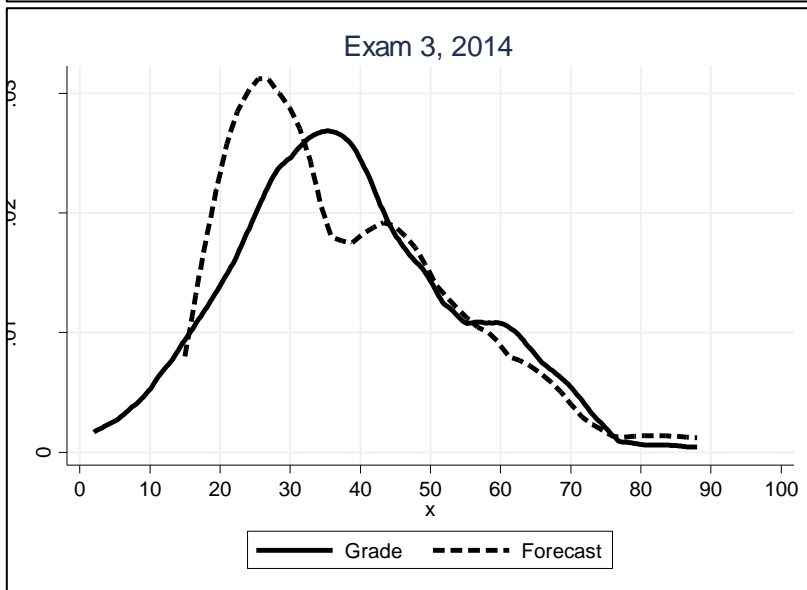

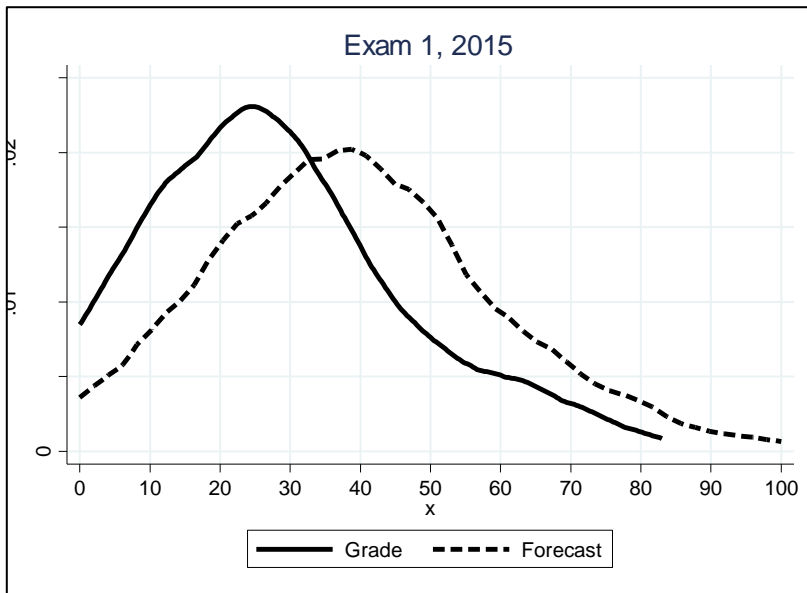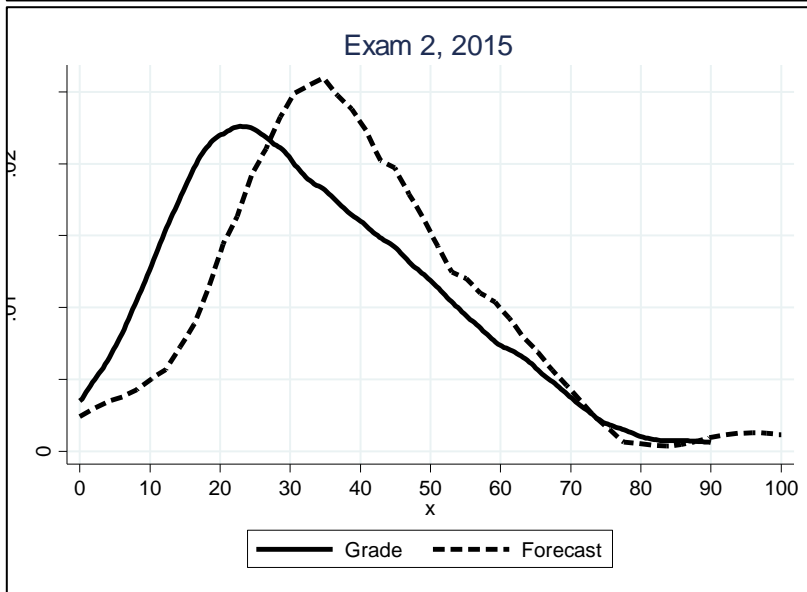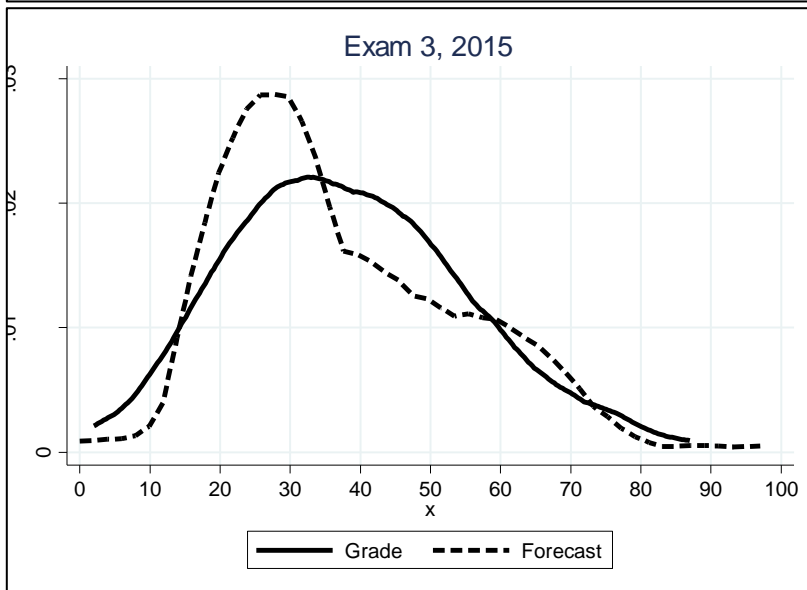

Supplement: Supplementary file 1 [file DataSheet1.PDF]
